# Deep Neural Networks Improve Radiologists’ Performance in Breast Cancer Screening

**DOI:** 10.1109/TMI.2019.2945514

**Published:** 2019-10-07

**Authors:** Nan Wu, Jason Phang, Jungkyu Park, Yiqiu Shen, Zhe Huang, Masha Zorin, Stanisław Jastrzębski, Thibault Févry, Joe Katsnelson, Eric Kim, Stacey Wolfson, Ujas Parikh, Sushma Gaddam, Leng Leng Young Lin, Kara Ho, Joshua D. Weinstein, Beatriu Reig, Yiming Gao, Hildegard Toth, Kristine Pysarenko, Alana Lewin, Jiyon Lee, Krystal Airola, Eralda Mema, Stephanie Chung, Esther Hwang, Naziya Samreen, S. Gene Kim, Laura Heacock, Linda Moy, Kyunghyun Cho, Krzysztof J. Geras

**Affiliations:** Center for Data Science, New York University, New York, NY 10011 USA.; Center for Data Science, New York University, New York, NY 10011 USA.; Center for Data Science, New York University, New York, NY 10011 USA.; Center for Data Science, New York University, New York, NY 10011 USA.; Center for Data Science, New York University, New York, NY 10011 USA.; NYU Courant Institute of Mathematical Sciences, New York University, New York, NY 10011 USA. She is now with the Department of Computer Science and Technology, University of Cambridge, Cambridge CB3 0FD, U.K.; Faculty of Mathematics and Information Technologies, Jagiellonian University, 30-348 Kraków, Poland.; Center for Data Science, New York University, New York, NY 10011 USA.; Department of Radiology, School of Medicine, New York University, New York, NY 10016 USA.; Department of Radiology, School of Medicine, New York University, New York, NY 10016 USA.; Department of Radiology, School of Medicine, New York University, New York, NY 10016 USA.; Department of Radiology, School of Medicine, New York University, New York, NY 10016 USA.; Department of Radiology, School of Medicine, New York University, New York, NY 10016 USA.; Department of Radiology, School of Medicine, New York University, New York, NY 10016 USA.; SUNY Downstate College of Medicine, New York, NY 11203 USA.; Department of Radiology, School of Medicine, New York University, New York, NY 10016 USA.; Department of Radiology, School of Medicine, New York University, New York, NY 10016 USA, and also with the Perlmutter Cancer Center, NYU Langone Health, New York, NY 10016 USA.; Department of Radiology, School of Medicine, New York University, New York, NY 10016 USA, and also with the Perlmutter Cancer Center, NYU Langone Health, New York, NY 10016 USA.; Department of Radiology, School of Medicine, New York University, New York, NY 10016 USA, and also with the Perlmutter Cancer Center, NYU Langone Health, New York, NY 10016 USA.; Department of Radiology, School of Medicine, New York University, New York, NY 10016 USA, and also with the Perlmutter Cancer Center, NYU Langone Health, New York, NY 10016 USA.; Department of Radiology, School of Medicine, New York University, New York, NY 10016 USA, and also with the Perlmutter Cancer Center, NYU Langone Health, New York, NY 10016 USA.; Department of Radiology, School of Medicine, New York University, New York, NY 10016 USA, and also with the Perlmutter Cancer Center, NYU Langone Health, New York, NY 10016 USA.; Department of Radiology, School of Medicine, New York University, New York, NY 10016 USA.; Department of Radiology, School of Medicine, New York University, New York, NY 10016 USA.; Department of Radiology, School of Medicine, New York University, New York, NY 10016 USA.; Department of Radiology, School of Medicine, New York University, New York, NY 10016 USA.; Department of Radiology, School of Medicine, New York University, New York, NY 10016 USA.; Department of Radiology, School of Medicine, New York University, New York, NY 10016 USA, with the Perlmutter Cancer Center, NYU Langone Health, New York, NY 10016 USA, and also with the Center for Advanced Imaging Innovation and Research, NYU Langone Health, New York, NY 10016 USA.; Department of Radiology, School of Medicine, New York University, New York, NY 10016 USA, and also with the Perlmutter Cancer Center, NYU Langone Health, New York, NY 10016 USA.; Department of Radiology, School of Medicine, New York University, New York, NY 10016 USA, with the Perlmutter Cancer Center, NYU Langone Health, New York, NY 10016 USA, and also with the Center for Advanced Imaging Innovation and Research, NYU Langone Health, New York, NY 10016 USA.; Center for Data Science, New York University, New York, NY 10011 USA, and also with the Courant Institute of Mathematical Sciences, New York University, New York, NY 10012 USA.; Department of Radiology, School of Medicine, New York University, New York, NY 10016 USA, with the Center for Data Science, New York University, New York, NY 10011 USA, and also with Center for Advanced Imaging Innovation and Research, NYU Langone Health, New York, NY 10016 USA

**Keywords:** Deep learning, deep convolutional neural networks, breast cancer screening, mammography

## Abstract

We present a deep convolutional neural network for breast cancer screening exam classification, trained, and evaluated on over 200 000 exams (over 1 000 000 images). Our network achieves an AUC of 0.895 in predicting the presence of cancer in the breast, when tested on the screening population. We attribute the high accuracy to a few technical advances. 1) Our network’s novel two-stage architecture and training procedure, which allows us to use a high-capacity patch-level network to learn from pixel-level labels alongside a network learning from macroscopic breast-level labels. 2) A custom ResNet-based network used as a building block of our model, whose balance of depth and width is optimized for high-resolution medical images. 3) Pretraining the network on screening BI-RADS classification, a related task with more noisy labels. 4) Combining multiple input views in an optimal way among a number of possible choices. To validate our model, we conducted a reader study with 14 readers, each reading 720 screening mammogram exams, and show that our model is as accurate as experienced radiologists when presented with the same data. We also show that a hybrid model, averaging the probability of malignancy predicted by a radiologist with a prediction of our neural network, is more accurate than either of the two separately. To further understand our results, we conduct a thorough analysis of our network’s performance on different subpopulations of the screening population, the model’s design, training procedure, errors, and properties of its internal representations. Our best models are publicly available at https://github.com/nyukat/breast_cancer_classifier.

## I. Introduction

BREAST cancer is the second leading cancer-related cause of death among women in the US. In 2014, over 39 million screening and diagnostic mammography exams were performed in the US. It is estimated that in 2015 232,000 women were diagnosed with breast cancer and approximately 40,000 died from it [1]. Although mammography is the only imaging test that has reduced breast cancer mortality [2]–[4], there has been discussion regarding the potential harms of screening, including false positive recalls and associated false positive biopsies. The vast majority of the 10–15% of women asked to return following an inconclusive screening mammogram undergo another mammogram and/or ultrasound for clarification. After the additional imaging exams, many of these findings are determined as benign and only 10–20% are recommended to undergo a needle biopsy for further work-up. Among these, only 20–40% yield a diagnosis of cancer [5]. Evidently, there is an unmet need to shift the balance of routine breast cancer screening towards more benefit and less harm.

Traditional computer-aided detection (CAD) in mammography is routinely used by radiologists to assist with image interpretation, despite multicenter studies showing these CAD programs do not improve their diagnostic performance [6]. These programs typically use handcrafted features to mark sites on a mammogram that appear distinct from normal tissue. The radiologist decides whether to recall these findings, determining clinical significance and actionability. Recent developments in deep learning [7]—in particular, deep convolutional neural networks (CNNs) [8]–[12]—open possibilities for creating a new generation of CAD-like tools.

This paper makes several technical contributions towards the goal of developing neural networks to support radiologists in interpreting breast cancer screening exams. (i) We introduce a novel two-stage neural network for incorporating global and local information with an appropriate training procedure. This allowed us to use a very high-capacity patch-level network to learn from pixel-level labels alongside a network learning from macroscopic breast-level labels. With this strategy, our model not only achieves a human-competitive performance but also produces interpretable heatmaps indicating locations of suspicious findings. Additionally, we show the utility of pixel-level labels even in a regime where we have a lot of image-level labels. (ii) We demonstrate the feasibility of training and evaluating the network with over 1,000,000 high-resolution mammographic images–an extremely large data set in medical imaging, not just for breast cancer screening. This has a significant value in both informing future research design priorities as well as showing a proof-of-concept and proof-of-value of this approach. We further perform a careful error analysis of our predictions, and identify patterns that our network was incapable of capturing, which will inform future architecture designs. (iii) To use as a building block of our network, we propose a novel variant of a ResNet specifically designed for medical imaging, which has a balance of depth and width that allows the model to process a very large image while maintaining reasonable memory consumption. (iv) We evaluate the utility of pretraining the network using a related task with a more noisy outcome (screening BI-RADS classification) and find it to be a very important part of the pipeline that markedly improves the performance of our models. This is of particular significance in medical imaging where most data sets are small. (v) We evaluate a number of ways to combine information from different mammographic views within a single neural network. The results of this analysis are also of value to a broader audience–including radiologists, particularly pertaining to the margin in performance between models trained on a subset of the views. We are not aware of any prior analysis like this, even though it is common for medical imaging tasks to have multiple inputs. (vi) We have made the code and weights of our best models available at https://github.com/nyukat/breast_cancer_classifier. With this contribution, research groups that are working on improving screening mammography, who may not have access to a large training dataset like ours, will be able to directly use our model in their research or use our pretrained weights as an initialization to train models with less data. By making our models public, we invite other groups to validate our results and test their robustness to shifts in the data distribution.

## II. Data

**Fig. 1.**
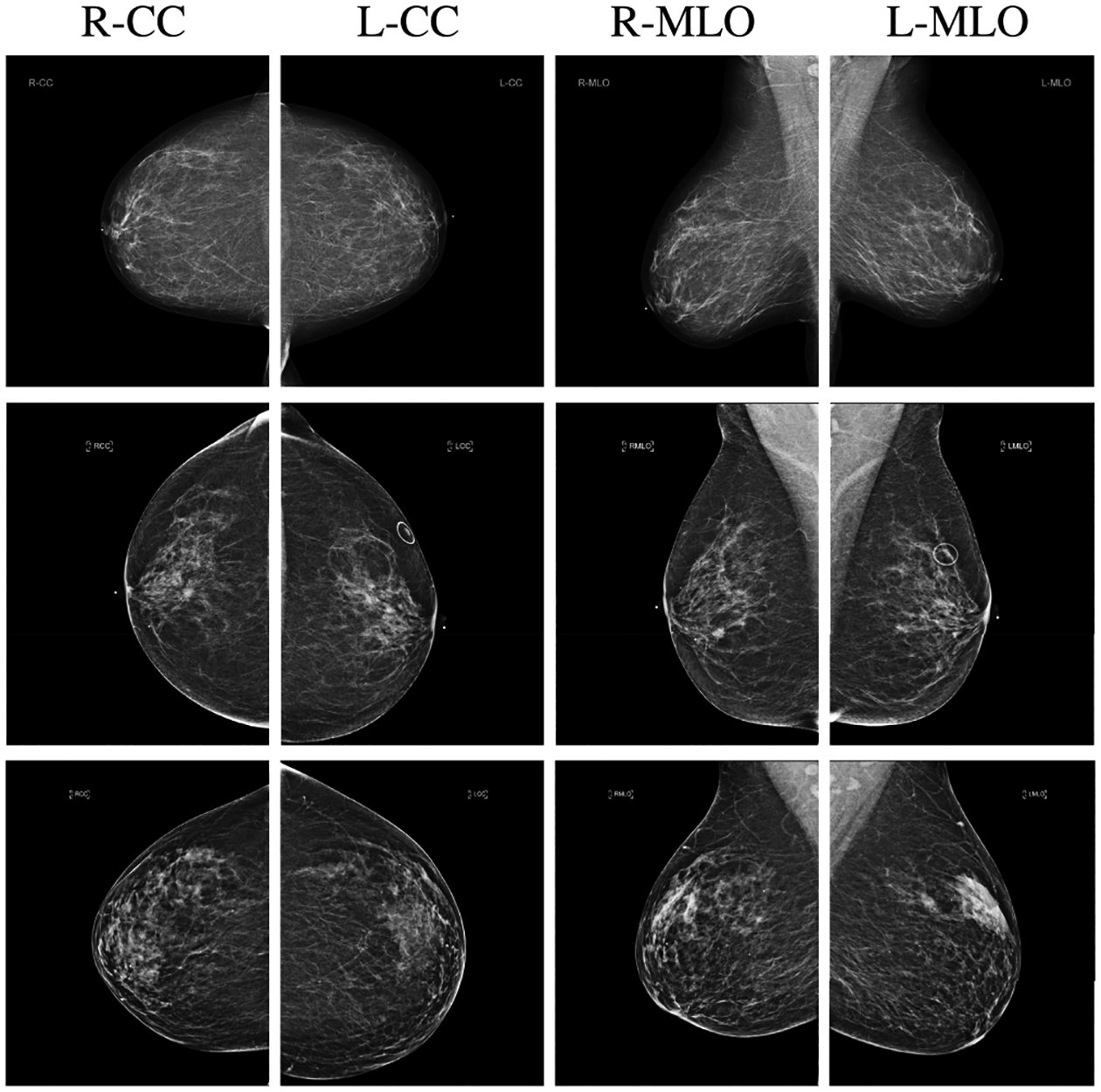
Examples of breast cancer screening exams. First row: both breasts without any findings; second row: left breast with no findings and right breast with a malignant finding; third row: left breast with a benign finding and right breast with no findings.

Our retrospective study was approved by our institutional review board and was compliant with the Health Insurance Portability and Accountability Act. Informed consent was waived. This dataset^1^ is a larger and more carefully curated version of a dataset used in our earlier work [14], [15]. The dataset includes 229,426 digital screening mammography exams (1,001,093 images) from 141,473 patients. Each exam contains at least four images,^2^ corresponding to the four standard views used in screening mammography: R-CC (right craniocaudal), L-CC (left craniocaudal), R-MLO (right mediolateral oblique) and L-MLO (left mediolateral oblique). The images in the dataset are coming from four types of scanners: Mammomat Inspiration (22.81%), Mammomat Novation DR (12.65%), Lorad Selenia (40.92%) and Selenia Dimensions (23.62%). A few examples of exams are shown in Fig. 1.

To extract labels indicating whether each breast of the patient was found to have malignant or benign findings at the end of the diagnostic pipeline, we relied on pathology reports from biopsies. We have 5,832 exams with at least one biopsy performed within 120 days of the screening mammogram. Among these, biopsies confirmed malignant findings for 985 (8.4%) breasts and benign findings for 5,556 (47.6%) breasts. 234 (2.0%) breasts had both malignant and benign findings. For the remaining screening exams that were not matched with a biopsy, we assigned labels corresponding to the absence of malignant and benign findings in both breasts.

**Fig. 2.**
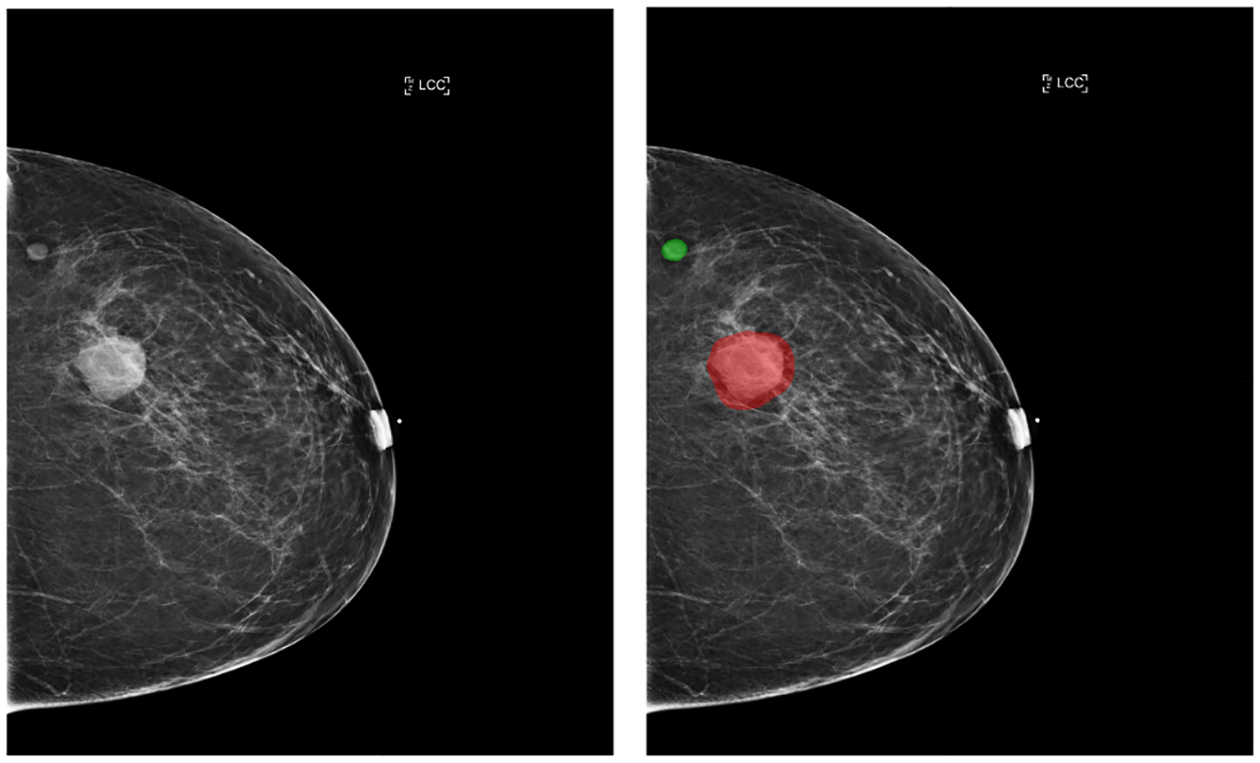
An example of a segmentation performed by a radiologist. Left: the original image. Right: the image with lesions requiring a biopsy highlighted. The malignant finding is highlighted with red and benign finding with green.

For all exams matched with biopsies, we asked a group of radiologists (provided with the corresponding pathology reports) to retrospectively indicate the location of the biopsied lesions at a pixel level. An example of such a segmentation is shown in Fig. 2. We found that approximately 32.8% of exams were mammographically occult, i.e., the lesions that were biopsied were not visible on mammography, even retrospectively, and were identified using other imaging modalities: ultrasound or MRI. See Table I for more details.

## III. Deep CNNS for Cancer Classification

**Fig. 3.**
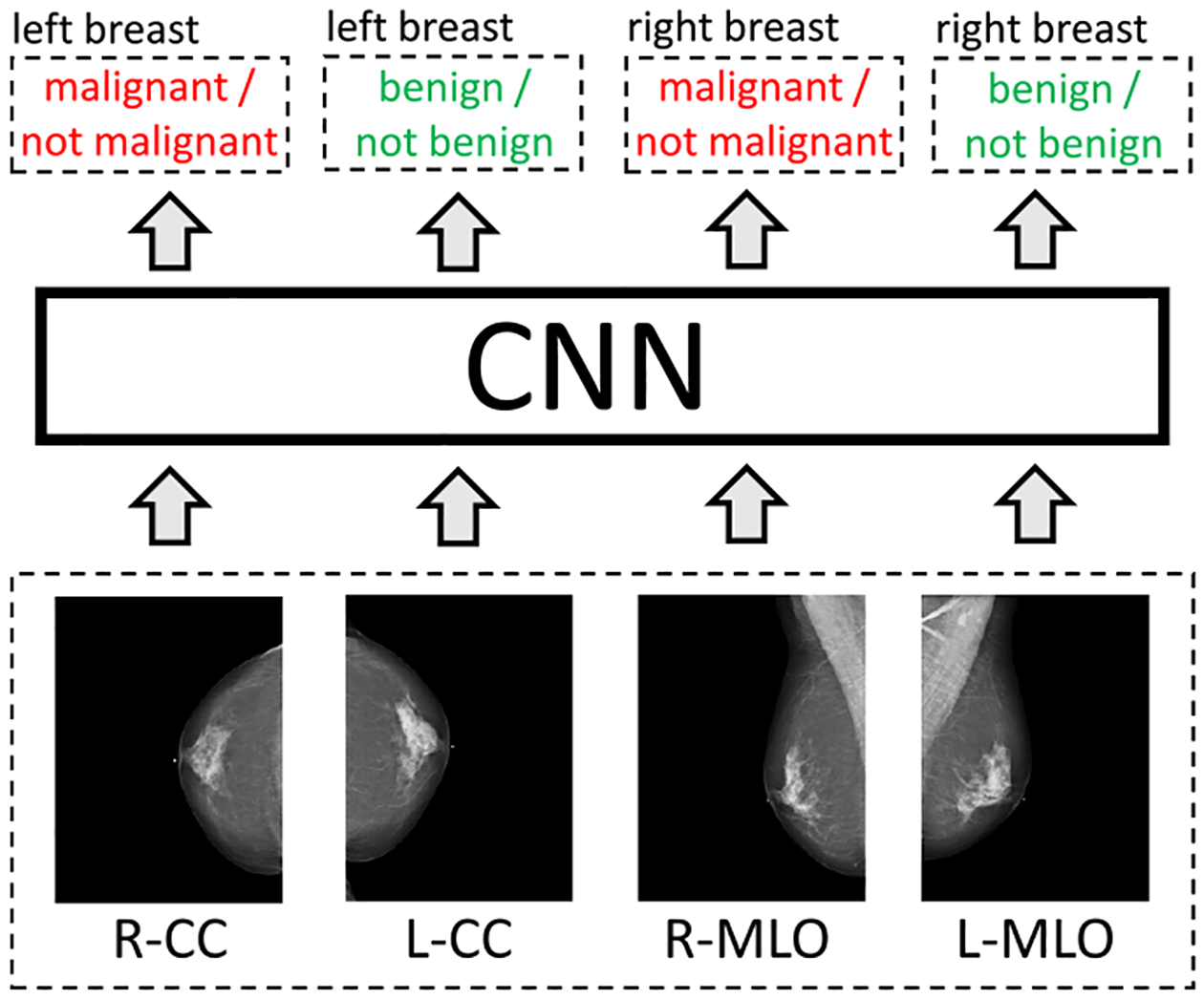
A schematic representation of how we formulated breast cancer exam classification as a learning task. The main task that we intend the model to learn is malignant/not malignant classification. The task of benign/not benign classification is used as an auxiliary task regularizing the network.

As some breasts contain both malignant and benign findings, we formulate breast cancer screening classification as a learning task using the multi-task learning framework [16]. That is, for each breast, we assign two binary labels: the absence/presence of malignant findings in a breast (denoted by *y*_R,m_ and *y*_L,m_), and the absence/presence of benign findings in a breast (denoted by *y*_R,b_ and *y*_L,b_). With left and right breasts, each exam has a total of four binary labels. Our goal is to produce four predictions corresponding to the four labels for each exam (denoted by ŷ_R,m_,ŷ_L,m_,ŷ_R,b_ and ŷ_L,b_). Although we are primarily interested in accurately predicting presence or absence of malignant findings, predicting the presence or absence of benign findings serves an important role of an auxiliary task regularizing learning the primary task. As input, we take four high-resolution images corresponding to the four standard screening mammography views (denoted by **x**_R−CC_, **x**_L−CC_, **x**_R−MLO_ and **x**_L−MLO_). We crop each image to a fixed size of 2677×1942 pixels for CC views and 2974×1748 pixels for MLO views.^3^ See Fig. 3 for a schematic representation.

## IV. Model Architecture and Training

We trained deep multi-view CNNs of four different architectures shown in Fig. 5, inspired by prior work of Geras *et al.* [14]. All of these networks consist of two core modules: (i) four view-specific columns, each based on the ResNet architecture [11] that output a fixed-dimension hidden representation for each mammography view, and (ii) two fully connected layers to map the computed hidden representations to the output predictions. The models differ in how the view-specific hidden representations from all views are aggregated to produce the final predictions. We considered the following variants.

1. The ‘view-wise’ model (Fig. 5(a)) concatenates L-CC and R-CC representations, and L-MLO and R-MLO representations. It makes separate predictions for CC and MLO views, which are averaged during inference.
2. The ‘image-wise’ model (Fig. 5(b)) makes a prediction for each of the four views independently. Corresponding predictions are averaged during inference.
3. The ‘side-wise’ (Fig. 5(c)) model first concatenates L-CC and L-MLO representations, and R-CC and R-MLO representations, then makes predictions for each breast separately.
4. The ‘joint’ model (Fig. 5(d)) concatenates the representations of all four views and jointly predicts malignant and benign findings for both breasts.

In all models, we used four ResNet-based 22-layer networks (*ResNet-22*) as columns computing a 256-dimension hidden representation vector of each view. In comparison to the standard ResNets, this network has a different balance of depth and width, which is adjusted to very high-resolution images. The details of the ResNet-22 network are in Section IV-A below. Experimentally, we found the ‘view-wise’ model to be the most accurate on the validation set in terms of the malignant/not malignant prediction task. Unless we explicitly specify otherwise, we report the results for this model.

### A. Single-View ResNet-22

**Fig. 4.**
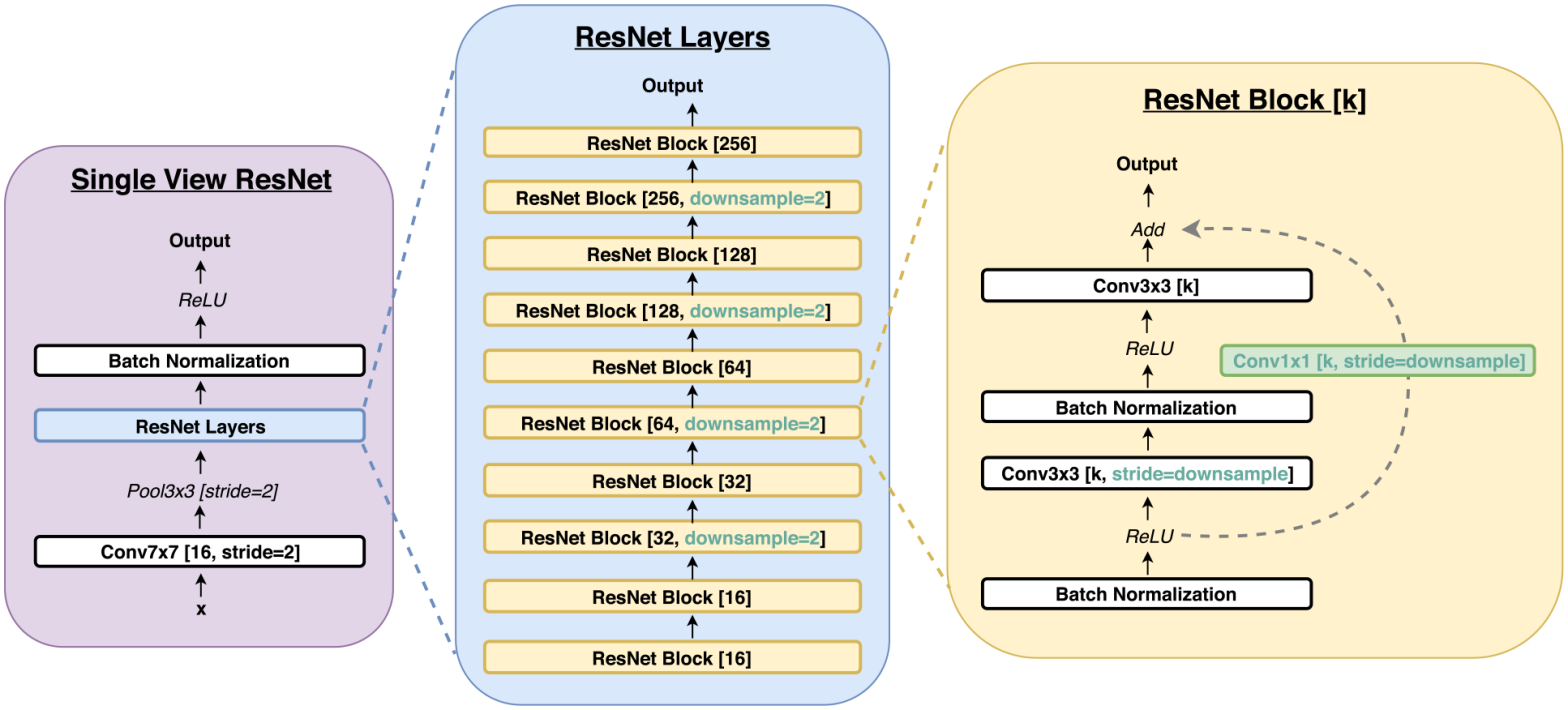
Architecture of single-view ResNet-22. The numbers in square brackets indicate the number of output channels, unless otherwise specified. **Left**: Overview of the single-view ResNet-22, which consists of a set of ResNet layers. **Center**: ResNet layers consist of a sequence of ResNet blocks with different downsampling and output channels. **Right**: ResNet blocks consist of two 3 × 3 convolutional layers, with interleaving ReLU and batch normalization operations, and a residual connection between input and output. Where no downsampling factor is specified for a ResNet block, the first 3 × 3 convolution layer has a stride of 1, and the 1 × 1 convolution operation for the residual is omitted.

**Fig. 5.**
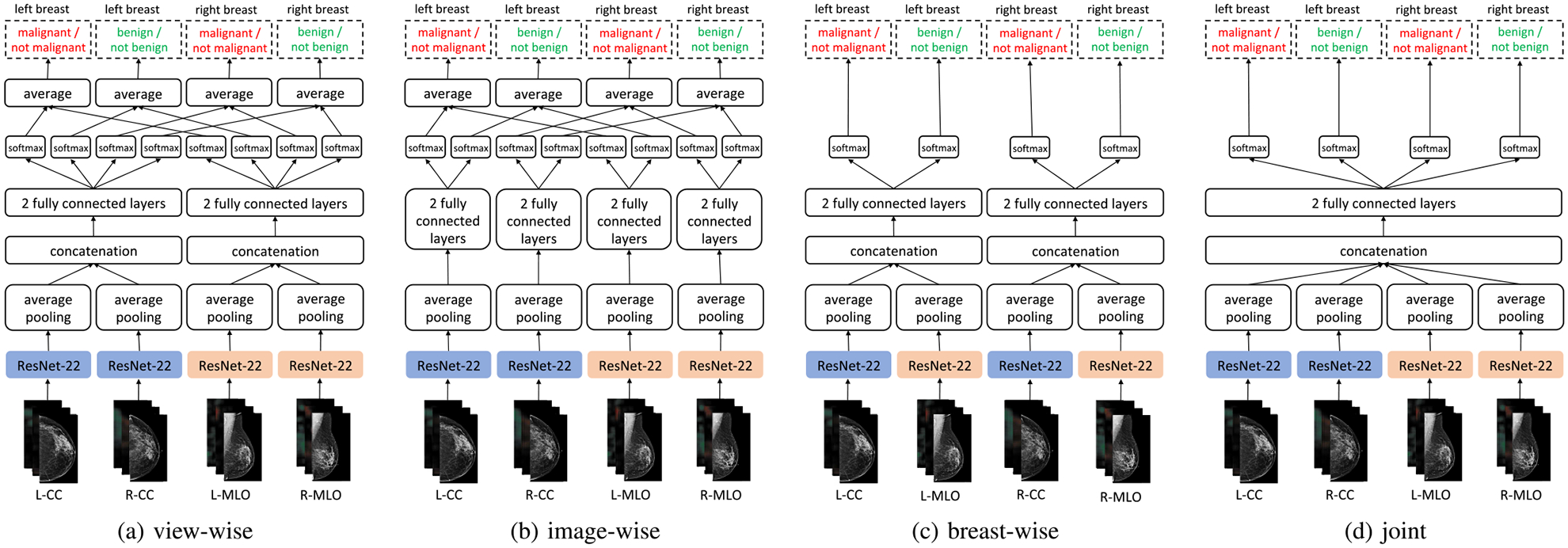
Four model variants for incorporating information across the four screening mammography views in an exam. All variants are constrained to have a total of 1,024 hidden activations between fully connected layers. The ‘view-wise’ model, which is the primary model used in our experiments, contains separate model branches for CC and MLO views–we average the predictions across both branches. The ‘image-wise’ model has a model branch for each image, and we similarly average the predictions. The ‘breast-wise’ model has separate branches per breast (left and right). The ‘joint’ model only has a single branch, operating on the concatenated representations of all four images. Average pooling in all models is averaging globally across spatial dimensions in all feature maps. When heatmaps (cf. Section IV-B) are added as additional channels to corresponding inputs, the first layers of the columns are modified accordingly.

The full architecture of ResNet-22 is shown in Fig. 4. We tied the weights for the L-CC and R-CC ResNets, as well as the L-MLO and R-MLO ResNets.^4^ Likewise, we flipped the L-CC and L-MLO images before feeding them to the model, so all breast images are rightward-oriented, allowing the shared ResNet weights to operate on similarly oriented images.

An intermediate output of each ResNet is a *H* × *W* × 256-dimensional tensor where *H* and *W* are downsampled for from the original input size, with *H* = 42 *W* = 31 for the CC view, and *H* = 47 and *W* = 28 for MLO view. We average-pool this represenation across the spatial dimensions to obtain a 256-dimension hidden representation vector for each view. For reference, we show the dimensions of the hidden activations after each major layer of the ResNet-22 in Table II.

The primary consideration in adapting the standard ResNets for mammograms is the need to process very high resolution images, without prior downsampling–fitting the forward pass and gradient computation within GPU memory. In addition, each processed minibatch needs to be sufficiently large for the model training to be well conditioned. For instance, we found that batch normalization adversely affects training for minibatch sizes smaller than four. We make several changes to create our ResNet-22. First, because the hidden representations at the lowest layers have undergone the least amount of downsampling and are thus the largest in size, we set the first convolutional layer to have relatively fewer channels: 16 compared to 64 in the standard ResNet models. To compensate, our model has 5 ResNet blocks compared to 4 in standard ResNets. As each ResNet block doubles the number of channels, our final hidden representation has 256 channels, compared to 512 in the case of standard ResNet models. Effectively, we increase the capacity across channels later in the model, trading off higher resolutions and fewer channels early on with smaller hidden represensentions and more channels later in the model. Lastly, whereas in standard ResNet models the classification layer is applied directly after global average pooling, in our model, we additionally apply two fully-connected layers before the classification layer. We do this in order to allow more complex interactions between different views.

#### 1) Training and Inference:

We trained the whole model using the Adam optimization algorithm [17], using a learning rate of 10^−5^ and a minibatch of size 4. We applied L_2_ regularization to our model weights with a coefficient of 10^−4.5^. The model has 6,132,592 trainable parameters (6,135,728 when using the heatmaps as described in Section IV-B, the only difference between both architectures is the size of the kernel in the first convolutional layer to accommodate the difference in the number of input channels). On an Nvidia V100 GPU, the model takes about 12 hours to train to the best validation performance (24 hours when using the heatmaps). A significant amount of training overhead is associated with the time to load and augment the high resolution mammography images. Details about data augmentation are in Section III in the Supplementary Material.

Only a small fraction of the exams in our training set contain images of biopsied breasts. Learning with data uniformly sampled from the training set would be very slow as the model would see few positive examples per epoch. To alleviate this issue, within each training epoch, the model was shown all exams with biopsies in the training set (4,844 exams) but only a random subset of an equal number of exams without biopsies (also 4,844 exams). We early-stopped the training when the average of the validation AUCs over the four prediction tasks did not improve for 20 epochs. We then selected the version of the model with the best validation AUC as our final model candidate (we show the training and validation curve for one image-only model and one image-and-heatmaps model in Section II-A in the Supplementary Material).

In preliminary experiments we noticed that when training the view-wise model, optimizing the prediction for each view separately leads to better generalization. Therefore, although at inference time the prediction for each breast is computed as an average of predictions for both views of that breast, the model is actually trained to optimize the loss, which treats the predictions for the two views separately. That is, the predictions for each target (as defined in Section III) are computed as

$${\hat{y}}_{\text{R},\text{m}}\left(x_{\text{R}-\text{CC}},x_{\text{L}-\text{CC}},x_{\text{R}-\text{MLO}},x_{\text{L}-\text{MLO}}\right)=\frac{1}{2}{\hat{y}}_{\text{R},\text{m}}^{\text{CC}}\left(x_{\text{R}-\text{CC}},x_{\text{L}-\text{CC}}\right)+\frac{1}{2}{\hat{y}}_{\text{R},\text{m}}^{\text{MLO}}\left(x_{\text{R}-\text{MLO}},x_{\text{L}-\text{MLO}}\right),$$

$${\hat{y}}_{\text{R},\text{b}}\left(x_{\text{R}-\text{CC}},x_{\text{L}-\text{CC}},x_{\text{R}-\text{MLO}},x_{\text{L}-\text{MLO}}\right)=\frac{1}{2}{\hat{y}}_{\text{R},\text{b}}^{\text{CC}}\left(x_{\text{R}-\text{CC}},x_{\text{L}-\text{CC}}\right)+\frac{1}{2}{\hat{y}}_{\text{R},\text{b}}^{\text{MLO}}\left(x_{\text{R}-\text{MLO}},x_{\text{L}-\text{MLO}}\right),$$

$${\hat{y}}_{\text{L},\text{m}}\left(x_{\text{R}-\text{CC}},x_{\text{L}-\text{CC}},x_{\text{R}-\text{MLO}},x_{\text{L}-\text{MLO}}\right)=\frac{1}{2}{\hat{y}}_{\text{L},\text{m}}^{\text{CC}}\left(x_{\text{R}-\text{CC}},x_{\text{L}-\text{CC}}\right)+\frac{1}{2}{\hat{y}}_{\text{L},\text{m}}^{\text{MLO}}\left(x_{\text{R}-\text{MLO}},x_{\text{L}-\text{MLO}}\right),$$

$${\hat{y}}_{\text{L},\text{b}}\left(x_{\text{R}-\text{CC}},x_{\text{L}-\text{CC}},x_{\text{R}-\text{MLO}},x_{\text{L}-\text{MLO}}\right)=\frac{1}{2}{\hat{y}}_{\text{L},\text{b}}^{\text{CC}}\left(x_{\text{R}-\text{CC}},x_{\text{L}-\text{CC}}\right)+\frac{1}{2}{\hat{y}}_{\text{L},\text{b}}^{\text{MLO}}\left(x_{\text{R}-\text{MLO}},x_{\text{L}-\text{MLO}}\right),$$

while the training loss is computed as

$$L\left(y_{\text{R},\text{m}},y_{\text{L},\text{m}},y_{\text{R},\text{m}},y_{\text{L},\text{m}},x_{\text{R}-\text{CC}},x_{\text{L}-\text{CC}},x_{\text{R}-\text{MLO}},x_{\text{L}-\text{MLO}}\right)=l\left(y_{\text{R},\text{m}},{\hat{y}}_{\text{R},\text{m}}^{\text{CL}}\left(x_{\text{R}-\text{CC}},x_{\text{L}-\text{CC}}\right)\right)+l\left(y_{\text{R},\text{m}},{\hat{y}}_{\text{R},\text{m}}^{\text{MLO}}\left(x_{\text{R}-\text{MLO}},x_{\text{L}-\text{MLO}}\right)\right)+l\left(y_{\text{R},\text{b}},{\hat{y}}_{\text{R},\text{b}}^{\text{CC}}\left(x_{\text{R}-\text{CC}},x_{\text{L}-\text{CC}}\right)\right)+l\left(y_{\text{R},\text{b}},{\hat{y}}_{\text{R},\text{b}}^{\text{MLO}}\left(x_{\text{R}-\text{MLO}},x_{\text{L}-\text{MLO}}\right)\right)+l\left(y_{\text{L},\text{m}},{\hat{y}}_{\text{L},\text{m}}^{\text{CC}}\left(x_{\text{R}-\text{CC}},x_{\text{L}-\text{CC}}\right)\right)+l\left(y_{\text{L},\text{m}},{\hat{y}}_{\text{L},\text{m}}^{\text{MLO}}\left(x_{\text{R}-\text{MLO}},x_{\text{L}-\text{MLO}}\right)\right)+l\left(y_{\text{L},\text{b}},{\hat{y}}_{\text{L},\text{b}}^{\text{CC}}\left(x_{\text{R}-\text{CC}},x_{\text{L}-\text{CC}}\right)\right)+l\left(y_{\text{L},\text{b}},{\hat{y}}_{\text{L},\text{b}}^{\text{MLO}}\left(x_{\text{R}-\text{MLO}},x_{\text{L}-\text{MLO}}\right)\right),$$

where l denotes binary cross-entropy.

The observation that when one of the two input modalities is more predictive than the other one, the network tends to ignore the less predictive modality is consistent with prior results [18]. In our experiments, we found that CC view is more predictive than MLO view (see Section I-C in the Supplementary Material).

### B. Auxiliary Patch-Level Classification Model and Heatmaps

The high resolution of the images and the limited memory of GPUs constrain us to use relatively shallow ResNets within our model when using full-resolution images as inputs. To further take advantage of the fine-grained detail in mammograms, we trained an auxiliary model to classify 256 × 256-pixel patches of mammograms, predicting presence or absence of malignant and benign findings in a given patch. The labels for these patches are determined based on the pixel-level segmentations of the corresponding mammograms produced by clinicians. We refer to this model as a *patch-level* model, in contrast to the *breast-level* model described in the section above which operates on images of the whole breast.

**Fig. 6.**
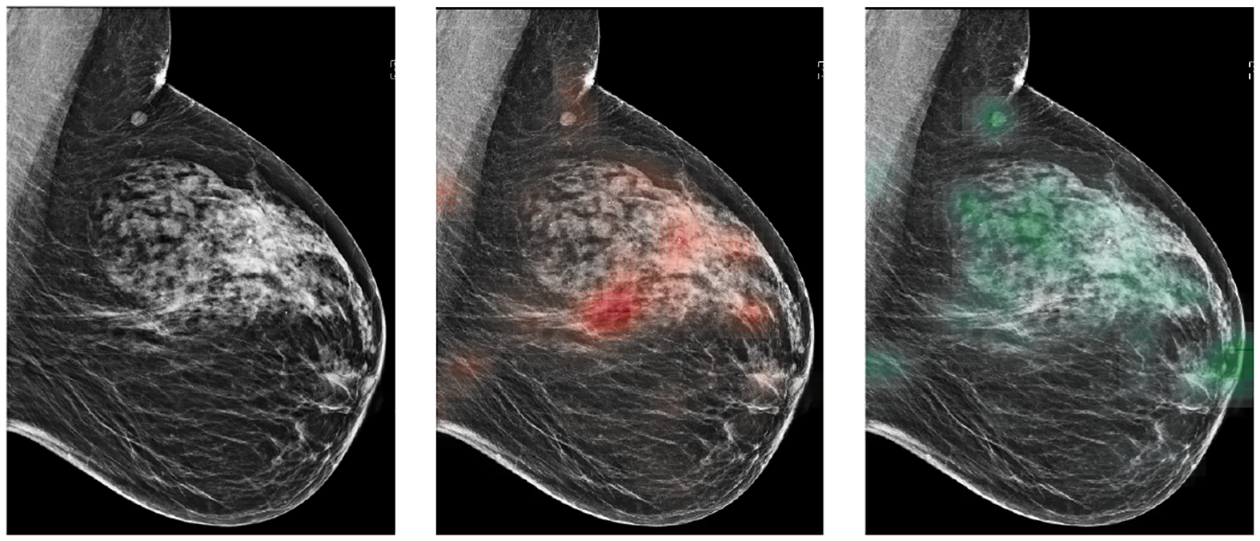
The original image (left), the ‘malignant’ heatmap over the image (middle) and the ‘benign’ heatmap over the image (right).

Subsequently, we apply this auxiliary network to the full resolution mammograms in a sliding window fashion to create two *heatmaps* for each image (an example in Fig. 6), one containing an estimated probability of a malignant finding for each pixel, and the other containing an estimated probability of a benign finding. Altogether, we obtain eight additional images: $x_{\text{R}-\text{CC}}^{\text{m}}$, $x_{\text{R}-\text{CC}}^{\text{b}}$, $x_{\text{L}-\text{CC}}^{\text{m}}$, $x_{\text{L}-\text{CC}}^{\text{b}}$, $x_{\text{R}-\text{MLO}}^{\text{m}}$, $x_{\text{R}-\text{MLO}}^{\text{b}}$, $x_{\text{L}-\text{MLO}}^{\text{m}}$, $x_{\text{L}-\text{MLO}}^{\text{b}}$. These patch classification heatmaps can be used as additional input channels to the breast-level model to provide supplementary fine-grained information. That is, the modified inputs to the network then are: $\left[x_{R-\text{CC}};x_{\text{R}-\text{CC}}^{\text{m}};x_{\text{R}-\text{CC}}^{\text{b}}\right]$, $\left[x_{\text{L}-\text{CC}};x_{\text{L}-\text{CC}}^{\text{m}};x_{\text{L}-\text{CC}}^{\text{b}}\right]$, $\left[x_{\text{R}-\text{MLO}};x_{\text{R}-\text{MLO}}^{\text{m}};x_{\text{R}-\text{MLO}}^{\text{b}}\right]$, $\left[x_{\text{L}-\text{MLO}};x_{\text{L}-\text{MLO}}^{\text{m}};x_{\text{L}-\text{MLO}}^{\text{b}}\right]$.

Using separate breast- and pixel-level models as described above differentiates our work from approaches which utilize pixel-level labels in a single differentiable network [19] or models based on the variations of R-CNN [20]. Our approach allows us to use a very deep auxiliary network at the patch level, as this network does not have to process the entire high-resolution image at once. Adding the heatmaps produced by the patch-level classifier as additional input channels allows the main classifier to get the benefit from pixel-level labels, while the heavy computation necessary to produce the pixel-level predictions does not need to be repeated each time an example is used for learning. We can also initialize the weights of the patch-level classifier using the weights of networks pretrained on large off-domain datasets such as ImageNet [21].^5^ Hereafter, we refer to the model using only mammogram images as the *image-only* model, and the model using mammogram images and the heatmaps as the *image-and-heatmaps* model.

### C. Pretraining on BI-RADS Classification

Because of the relatively small number of biopsied examples with benign or malignant labels we have available, we apply transfer learning to improve the robustness and performance of our models. Transfer learning involves reusing parts of a model pretrained on another task as a starting point for training the target model, taking advantage of the learned representations from the pretraining task.

For our model, we apply transfer learning from a network pretrained on a BI-RADS classification task, as in [14], which corresponds to predicting a radiologist’s assessment of a patient’s risk of having breast cancer based only on screening mammography. The three BI-RADS classes we consider are: BI-RADS Category 0 (“incomplete”), BI-RADS Category 1 (“normal”) and BI-RADS Category 2 (“benign”). The algorithm used to extract these labels is explained in [13]. Although these labels are more noisy than biopsy outcomes (being assessments of clinicians based on screening mammograms and not informed by a biopsy), compared to the 4,844 exams with biopsy-proven cancer labels in the training set, we have over 99,528 training examples with BI-RADS 0 and BI-RADS 2 labels. Neural networks have been shown to reach reasonable levels of performance even when trained with noisy labels [22], [23]. We use this property to transfer the information learned with BI-RADS labels to the cancer classification model. In fact, our experiments show that pretraining on BI-RADS classification contributes significantly to the performance of our model (see Section V-E).

**Fig. 7.**
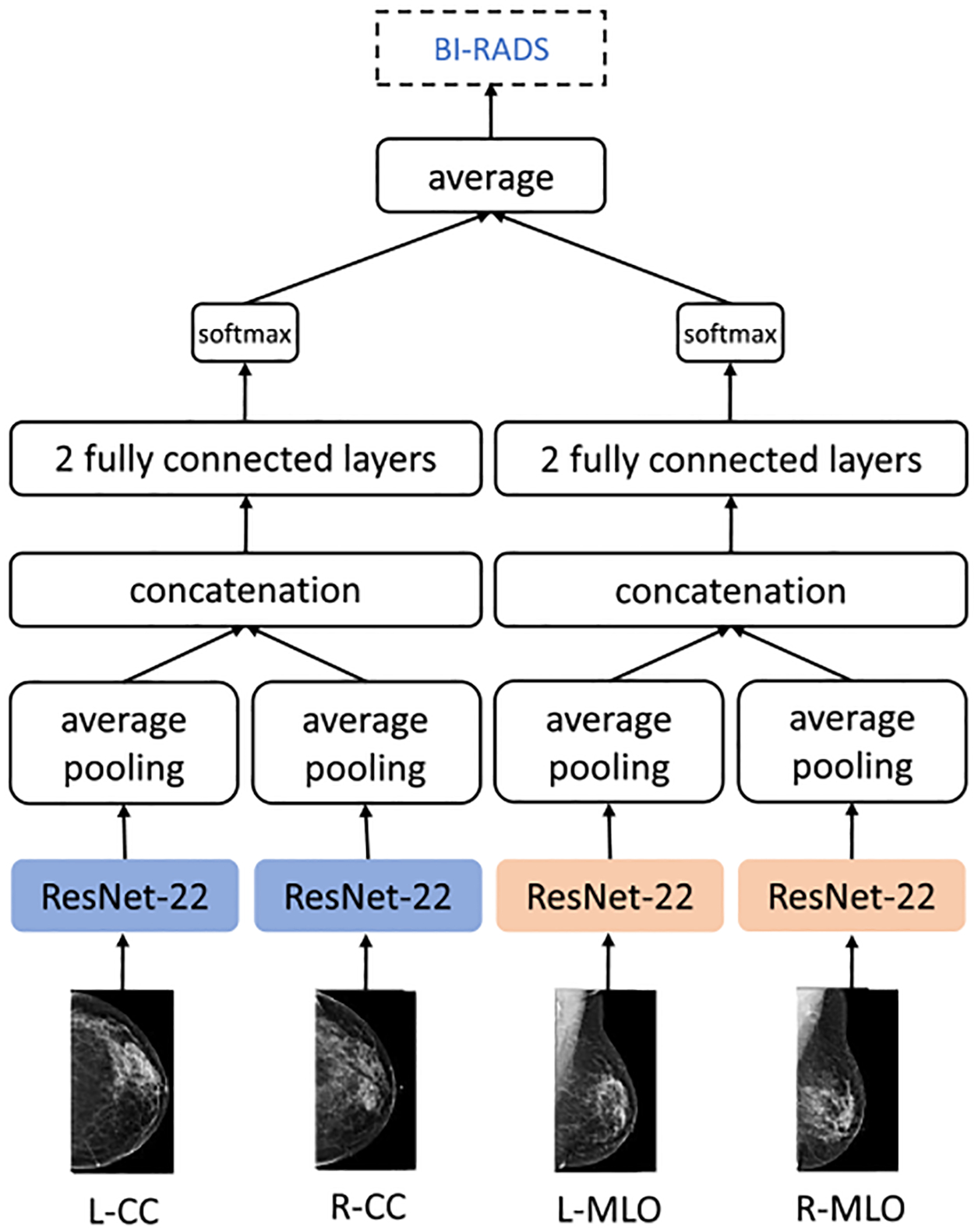
BI-RADS classification model architecture. The architecture is largely similar to the ‘view-wise’ cancer classification model variant, except that the output is a set of probability estimates over the three output classes. The model consists of four ResNet-22 columns, with weights shared within CC and MLO branches of the model.

The model we use for BI-RADS classification is shown in Fig. 7. It is similar to the ‘view-wise’ model architecture for cancer classification described in the *Model variants* section above, except that the output layer outputs probability estimates over three classes for a single label. We measured the performance of this model by averaging AUCs of 0-vs-other, 1-vs-other and 2-vs-other predictions on the validation set.

The rest of the training details (e.g. ResNet-22 architecture, optimizer hyperparameters) are identical to those of the cancer classification model, except that the model was trained with a minibatch size of 24 instead of 4. We early-stopped training based on validation AUCs after no improvement for 20 epochs, and initialized the ResNet-22 weights for the cancer classification model using the learned weights in the BI-RADS model. Where we used heatmaps as additional input channels, we duplicated the weights on the bottommost convolutional kernel such that the model can operate on inputs with three channels–the rest of the model is left unchanged. In our experimental results, we used a BI-RADS model trained for 111 epochs (326 hours on four Nvidia V100 GPUs), which obtained an averaged validation AUC of 0.748.

We emphasize here that we used the same train-validation-test splits for pretraining our BI-RADS classification model as in training our cancer classification model, so no data leakage across splits was possible.

## V. Experiments

In all experiments, we used the training set for optimizing parameters of our model and the validation set for tuning hyperparameters of the model and the training procedure. Unless otherwise specified, results were computed across the screening population. To obtain predictions for each test example, we apply random transformations to the input 10 times, apply the model to each of the 10 samples separately and then average the 10 predictions (details in Section III in the Supplementary Material).

To further improve our results, we employed the technique of model ensembling [24], wherein the predictions of several different models are averaged to produce the overall prediction of the ensemble. In our case, we trained five copies of each model with different random initializations of the weights in the fully connected layers, while the remaining weights are initialized with the weights of the model pretrained on BI-RADS classification. For each model, we report the results from a single network (mean and standard deviation across five random initializations) and from an ensemble.

### A. Test Populations

In the experiments below, we evaluate our model on several populations to test different hypotheses: (i) *screening population*, including all exams from the test set without subsampling; (ii) *biopsied subpopulation*, which is subset of the screening population, only including exams from the screening population containing breasts which underwent a biopsy; (iii) *reader study subpopulation*, which consists of the biopsied subpopulation and a subset of randomly sampled exams from the screening population without any findings.

### B. Evaluation Metrics

We evaluated our models primarily in terms of AUC (area under the ROC curve) for malignant/not malignant and benign/not benign classification tasks on the breast level. The model and readers’ responses on the subset for the reader study are evaluated in terms of AUC as well as precision-recall AUC (PRAUC), which are commonly used metrics in the evaluation of radiologists’ performance. ROC and PRAUC capture different aspects of performance of a predictive model. The ROC curve summarizes the trade-off between the true positive rate and false positive rate for a model using different probability thresholds. The precision-recall curve summarizes the trade-off between the true positive rate (recall) and the positive predictive value (precision) for a model using different probability thresholds.

### C. Screening Population

In this section we present the results on the screening population, which approximates the distribution of patients who undergo routine screening. Results across different model variants are shown in Table III. Overall, all four model variants achieve high and relatively similar AUCs. The ‘view-wise’ image-and-heatmaps ensemble, which is also architecturally most similar to the BI-RADS model used in the pretraining stage, performs the best in predicting malignant/not malignant, attaining an AUC of 0.895 on the screening population and 0.850 on the biopsied population. However, some of the other model variants do outperform the ‘view-wise’ ensemble for benign/not-benign prediction. Among the image-only models, the four model variants perform roughly comparably, though still consistently underperforming the image-andheatmaps models. The image-and-heatmaps models improve more strongly in malignant/not malignant classification than benign/not benign classification. We also find that ensembling is beneficial across all models, leading to a small but consistent increase in AUC.

Constructing an ensemble of the four model variants for the image-and-heatmaps model, with five randomly initialized models per variant, results in an AUC of 0.778 on benign/not benign prediction, and 0.899 on malignant/not malignant prediction on the screening population. Although this performance is superior to any individual model variant, running such a large ensemble of 20 separate models would be prohibitively expensive in practice.

The discrepancy in performance of our models between the malignant/not malignant the benign/not benign tasks can be largely explained by the fact that a larger fraction of benign findings than malignant findings are mammographically-occult (Table I). Additionally, there can be noise in the benign/not benign labels associated with radiologists’ confidence in their diagnoses. For the same exam, one radiologist might discard a finding as obviously not malignant without requesting a biopsy, while another radiologist might be more conservative and ask for a biopsy.

Using the validation set, we found that the ‘view-wise’ image-and-heatmaps model outperforms all other variants in terms of the average of AUCs for malignant/not malignant and benign/not benign prediction tasks. Unless otherwise specified, for both image-only and image-and-heatmaps model, we are referring to results based on the ‘view-wise’ model in the following sections.

### D. Biopsied Subpopulation

We show the results of our models evaluated only on the biopsied subpopulation, in the right half of Table III. Within our test set, this corresponds to 401 breasts: 339 with benign findings, 45 with malignant findings, and 17 with both. This subpopulation that underwent biopsy with at least one imaging finding differs markedly from the overall screening population, which consists of largely healthy individuals undergoing routine annual screening without recall for additional imaging or biopsy. Compared to the results on the screening population, AUCs on the biopsied population are markedly lower across all the model variants.

On the biopsied subpopulation, we observed a consistent difference between the performance of image-only and image-and-heatmaps models. The ensemble of image-and-heatmaps models performs best on both malignant/not malignant classification, attaining an AUC of 0.850, and on benign/not benign classification, attaining an AUC of 0.696. The markedly lower AUCs attained for the biopsied subpopulation, in comparison to the screening population, can be explained by the fact that exams that require a recall for diagnostic imaging and that subsequently need a biopsy are more challenging for both radiologists and our model.^6^

### E. Importance of Pretraining on BI-RADS Classification

In this section, we evaluate the benefit of the BI-RADS pretraining by comparing the performance of our models to cancer classification models trained without using weights from a pretrained BI-RADS model. Specifically, we train a set of cancer classification models by starting from entirely randomly initialized model weights.

The results are shown in Table III (marked with *). In every case, we see an improvement in performance from using weights of a model pretrained on BI-RAD classification, compared to randomly initializing the model weights and training from scratch. The improvement in performance from using pretrained weights tends to be larger for the image-only model compared to image-and-heatmaps models. We hypothesize that this is because the heatmaps already contain significant information pertaining to cancer classification, and hence the model can likely more quickly learn to make use of the heatmaps for cancer classification. In contrast, the image-only models rely entirely on the ResNets to effectively encode visual information for cancer classification, and therefore using the weights of a model pretrained for BI-RADS classification contributes significantly to the model performance.

## VI. Reader Study

To compare the performance of our image-and-heatmaps ensemble (hereafter referred to as *the model*) to human radiologists, we performed a reader study with 14 readers—12 attending radiologists at various levels of experience (between 2 and 25 years), a resident and a medical student—each reading 740 exams from the test set (1,480 breasts): 368 exams randomly selected from the biopsied subpopulation and 372 exams randomly selected from exams not matched with any biopsy. Exams were shuffled before being given to the readers. Readers were asked to provide a probability estimate of malignancy on a 0%−100% scale for each breast. As some breasts contain multiple suspicious findings, readers were asked to give their assessment of the most suspicious finding.

We used the first 20 exams as a practice set to familiarize readers with the format of the reader study–these were excluded from the analysis.^7^ On the remaining 720 exams, we evaluated the model’s and readers’ performance on malignancy classification. Among the 1,440 breasts, there are 62 breasts labeled as malignant and 356 breasts labeled as benign. In the breasts labeled as malignant, there are 21 masses, 26 calcifications, 12 asymmetries and 4 architectural distortions.^8,9^ In the breasts labeled as benign, the corresponding numbers of imaging findings are: 87, 102, 36 and 6.

**Fig. 8.**
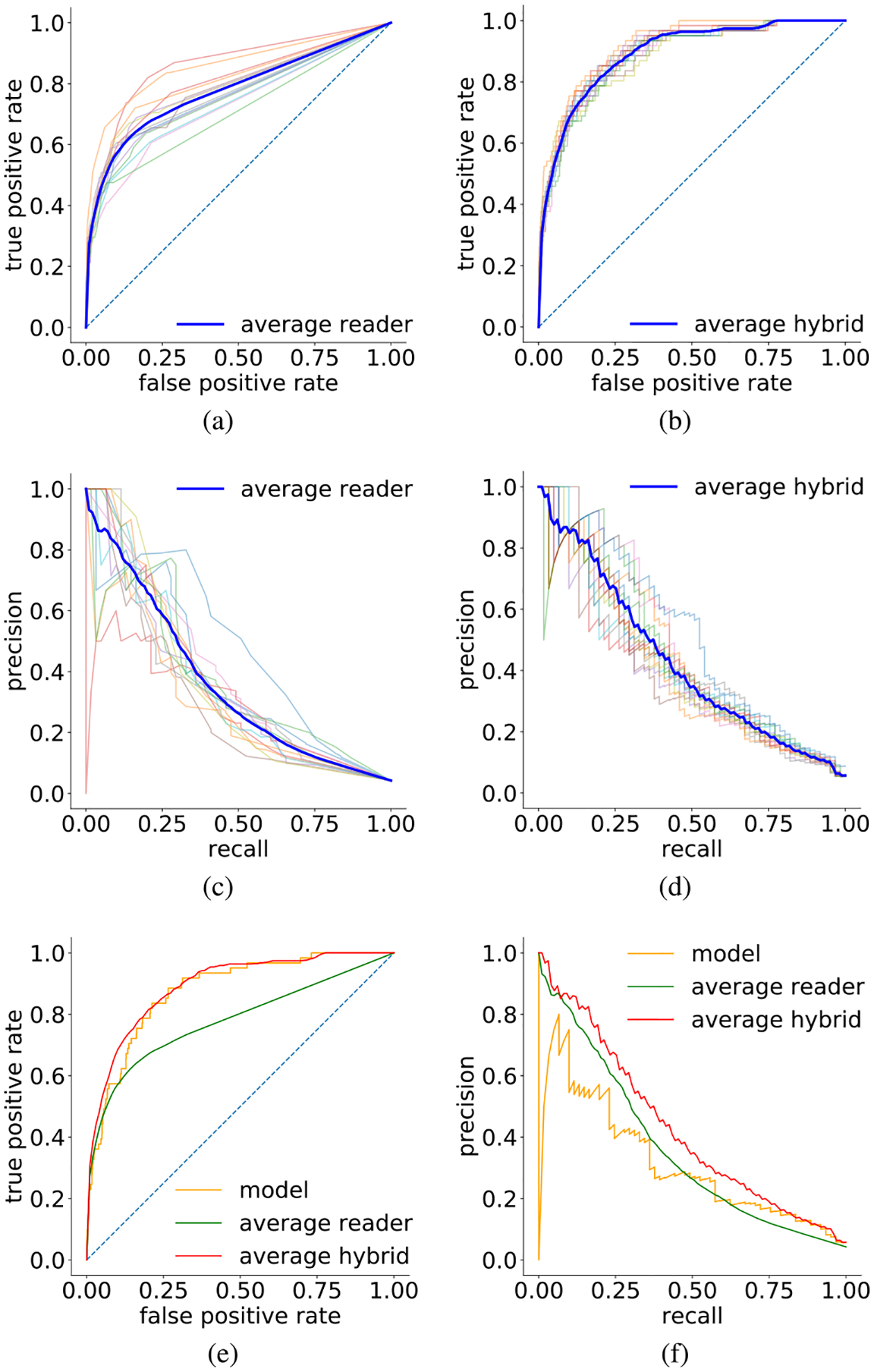
ROC curves [(a), (b), and (e)] and Precision-Recall curves [(c), (d), and (f)] on the subset of the test set used for the reader study. (a) and (c) curves for all 14 readers. Their average performance are highlighted in blue. (b) and (d) curves for hybrid of the image-andheatmaps ensemble with each single reader. Curve highlighted in blue indicates the average performance of all hybrids. (e) and (f) comparison among the image-and-heatmaps ensemble, average reader and average hybrid.

Our model achieved an AUC of 0.876 and PRAUC of 0.318. AUCs achieved by individual readers varied from 0.705 to 0.860 (mean: 0.778, std: 0.0435). PRAUCs for readers varied from 0.244 to 0.453 (mean: 0.364, std: 0.0496). Individual ROCs and precision-recall curves, along with their averages are shown in Fig. 8(a) and Fig. 8(c).

We also evaluated the accuracy of a human-machine hybrid, whose predictions are a linear combination of predictions of a radiologist and of the model–that is,

$${\hat{y}}_{\text{hybrid}}=\lambda {\hat{y}}_{\text{radiologist}}+(1-\lambda ){\hat{y}}_{\text{model}}.$$

**Fig. 9.**
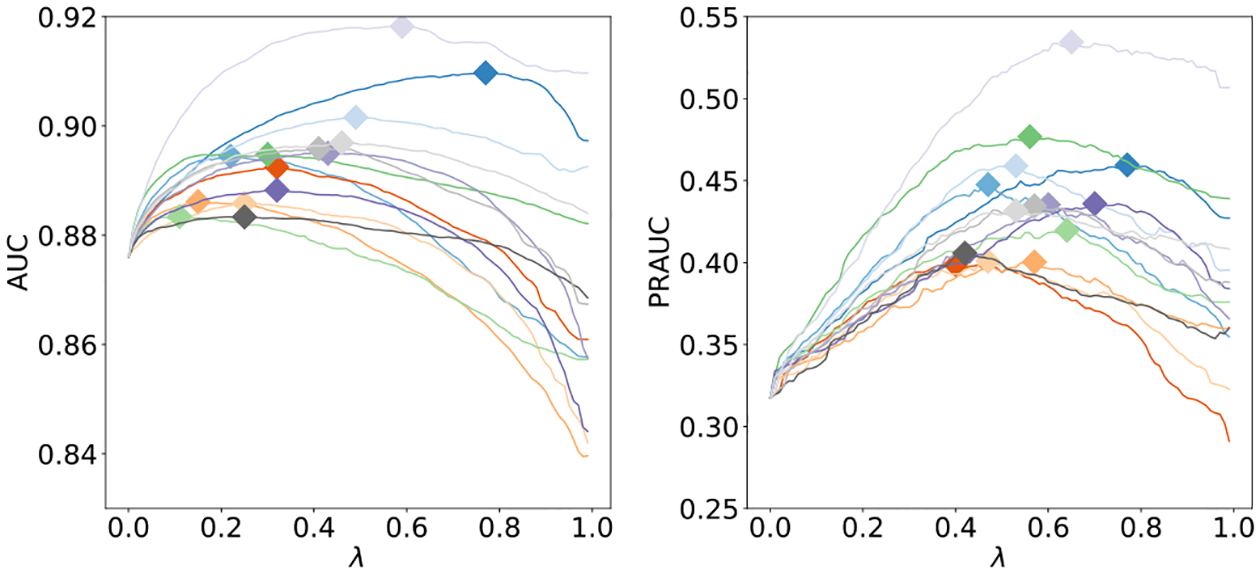
AUC (left) and PRAUC (right) as a function of λ ∈ [0, 1) for hybrids between each reader and our image-and-heatmaps ensemble. Each hybrid achieves the highest AUC/PRAUC for a different λ (marked with ◇).

For *λ* = 0.5^10^ (see Fig. 9 for the results for *λ* ∈ [0, 1)), hybrids between each reader and the model achieved an average AUC of 0.891 (std: 0.0109) and an average PRAUC of 0.431 (std: 0.0332) (cf. Fig. 8(b), Fig. 8(d)). These results suggest our model can be used as a tool to assist radiologists in reading breast cancer screening exams and that it captured different aspects of the task compared to experienced breast radiologists. A qualitative analysis comparing predictions made by our network and by the radiologists for specific exams can be found in Section I-G-1 in the Supplementary Material.

### A. Visualization of the Representation Learned by the Classifier

Additionally, we examined how the network represents the exams internally by visualizing the hidden representations learned by the best single image-and-heatmaps model, for exams in reader study subpopulation. We visualize two sets of activations: concatenated activations from the last layer of each of the four image-specific columns, and concatenated activations from the first fully connected layer in both CC and MLO model branches. Both sets of activations have 1,024 dimensions in total. We embed them into a two-dimensional space using UMAP [25] with the Euclidean distance.

**Fig. 10.**
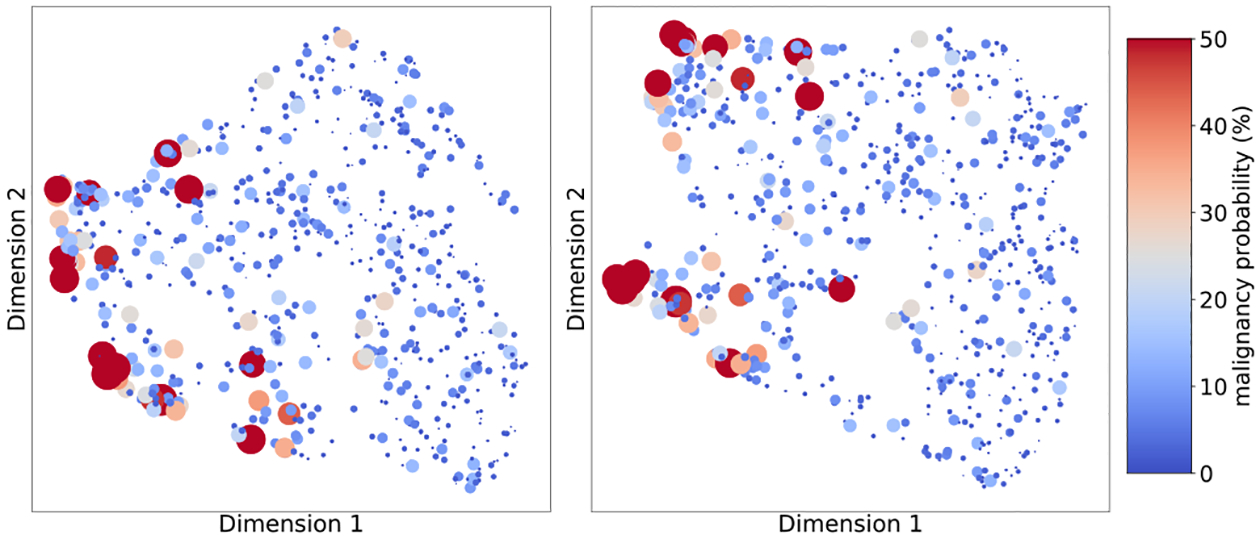
Two-dimensional UMAP projection of the activations computed by the network for the exams in the reader study. We visualize two sets of activations: (left) concatenated activations from the last layer of each of the four image-specific columns, and (right) concatenated activations from the first fully connected layer in both CC and MLO model branches. Each point represents one exam. Color and size of each point reflect the same information: probability of malignancy predicted by the readers (averaged over the two breasts and the 14 readers).

Fig. 10 shows the embedded points. Color and size of each point reflect the same information: the warmer and larger the point is, the higher the readers’ mean prediction of malignancy is. A score for each exam is computed as an average over predictions for the two breasts. We observe that exams classified as more likely to be malignant according to the readers are close to each other for both sets of activations. The fact that previously unseen exams with malignancies were found by the network to be similar further corroborates that our model exhibits strong generalization capabilities.

## VII. Related Work

Prior works approach the task of breast cancer screening exam classification in two paradigms. In one paradigm, only exam-level, breast-level or image-level labels are available. A CNN is first applied to each of the four standard views and the resulting feature vectors are combined to produce a final prediction [14]. This workflow can be further integrated with multi-task learning where radiological assessments, such as breast density, can be incorporated to model the confidence of the classification [26]. Other works formulate the breast cancer exam classification task as weakly supervised localization and produce a class activation map that highlights the locations of suspicious lesions [27]. Such formulations can be paired with multiple-instance learning where each spatial location is treated as a single instance and associated with a score that is correlated with the existence of a malignant finding [28].

In the second paradigm, pixel-level labels that indicate the location of benign or malignant findings are also provided to the classifier during training. The pixel-level labels enable training models derived from the R-CNN architecture [20] or models that divide the mammograms into smaller patches and train patch-level classifiers using the location of malignant findings [19], [29]–[32]. Some of these works directly aggregate outputs from the patch-level classifier to form an image-level prediction. A major limitation of such architectures is that information outside the annotated regions of interest will be neglected. Other works apply the patch-level classifier as a first level of feature extraction on top of which more layers are stacked and the entire model is then optimized jointly. A downside of this kind of architecture is the requirement for the whole model to fit in GPU memory for training, which limits the size of the minibatch used (usually to one), depth of the patch-level model and how densely the patch-level model is applied. Our work is most similar to the latter type of models utilizing pixel-level labels, however, our strategy uses a patch-level classifier for producing heatmaps as additional input channels to the breast-level classifier. While we forgo the ability to train the whole model end-to-end, the patch-level classifier can be significantly more powerful and can be densely applied across the original image. As a result, our model has the ability to learn both local features across the entire image as well as macroscopic features such as symmetry between breasts. For a more comprehensive review of prior work, refer to one of the recent reviews [33], [34].

A variety of results in terms of AUC for prediction of malignancy have been reported. The most comparable to our work are: [28] (0.86), [20] (0.95), [35] (0.81), [27] (0.91), [36] (0.84) and [37] (0.89). Unfortunately, although these results can serve as a rough estimate of model quality, comparing different methods based on these numbers would be misleading. Some authors do not discuss the design of their models [35]–[37], some evaluate their models on very small public datasets, InBreast [38] or DDSM [39], which are insufficient for a meaningful evaluation, while others used private datasets with populations of different distributions (on a spectrum between screening population and biopsied subpopulation), different quality of imaging equipment and even differently defined labels. By making the code and the weights of our model public, we seek to enable more direct comparisons to our work.

## VIII. Discussion and Conclusions

By leveraging a large training set with breast-level and pixel-level labels, we built a neural network which can accurately classify breast cancer screening exams. We attribute this success to the significant amount of computation encapsulated in the patch-level model, which was densely applied to the input images to form heatmaps as additional input channels to a breast-level model. It would be impossible to train this model in a completely end-to-end fashion with currently available hardware. Although our results are promising, we acknowledge that the test set used in our experiments is relatively small and our results require further clinical validation. We also acknowledge that although our network’s performance is stronger than that of the radiologists’ on the specific task in our reader study, this is not exactly the task that radiologists perform. Typically, screening mammography is only the first step in a diagnostic pipeline, with the radiologist making a final determination and decision to biopsy only after recall for additional diagnostic mammogram images and possible ultrasound. However, in our study, a hybrid model including both a neural network and expert radiologists outperformed either individually, suggesting that the use of such a model could improve radiologist sensitivity for breast cancer detection.

On the other hand, the design of our model is relatively simple. More sophisticated and accurate models are possible. Furthermore, the task we considered in this work, predicting whether the patient had a visible cancer at the time of the screening mammography exam, is the simplest possible among many tasks of interest. In addition to testing the utility of this model in real-time reading of screening mammograms, a clear next step would be predicting the development of breast cancer in the future–before it is even visible to a trained human eye.

## Figures and Tables

**TABLE I:** Number of Breasts With Malignant and Benign Findings Based on the Labels Extracted From the Pathology Reports, Broken Down According to Whether the Findings Were Visible or Occult

	malignant		benign	
	visible	occult	visible	occult
**training**	750	107	2,586	2,004
**validation**	51	15	357	253
**test**	54	8	215	141
**overall**	855 (86.8%)	130 (13.2%)	3,158 (56.84%)	2,398 (43.16%)

**TABLE II:** Dimensions of Feature Maps After Each Layer in Resnet-22, Shown as *H* × *W* × *D*. *D* Indicates the Number of Feature Maps, *H* and *W* Indicate Spatial Dimensions

	CC view	MLO view
Conv7×7	1339×971×16	1487×874×16
ResBlock 1	670×486×16	744×437×16
ResBlock 2	335×243×32	372×219×32
ResBlock 3	168×122×64	186×110×64
ResBlock 4	84×61×128	93×55×128
ResBlock 5	42×31×256	47×28×256

**TABLE III:** AUCS of Our Models on Screening and Biopsied Populations. All Models, Except the Ones Indicated With * Were Pretrained on BI-RADS Classification

		screening population				biopsied population			
		single		5x ensemble		single		5x ensemble	
		malignant	benign	malignant	benign	malignant	benign	malignant	benign
**image-only**	view-wise	0.827±0.008	0.731 ±0.004	0.840	0.743	0.781±0.006	0.673±0.003	0.791	0.682
	view-wise*	0.687±0.009	0.657±0.006	0.703	0.669	0.693±0.006	0.564±0.006	0.709	0.571
	image-wise	0.830±0.006	0.759±0.002	0.841	0.766	0.740±0.007	0.638±0.001	0.749	0.642
	breast-wise	0.821±0.012	0.757±0.002	0.836	0.768	0.726±0.009	0.639±0.002	0.738	0.645
	joint	0.822±0.008	0.737±0.004	0.831	0.746	0.780±0.006	0.682±0.001	0.787	0.688
**image-and-heatmaps**	view-wise	**0.886±0.003**	0.747±0.002	**0.895**	0.756	**0.843±0.004**	0.690±0.002	**0.850**	0.696
	view-wise*	0.856±0.007	0.701±0.004	0.868	0.708	0.828±0.008	0.633±0.006	0.841	0.640
	image-wise	0.875±0.001	**0.765±0.003**	0.885	0.774	0.812±0.001	0.653±0.003	0.821	0.658
	breast-wise	0.876±0.004	0.764±0.004	0.889	**0.779**	0.805±0.004	0.652±0.004	0.818	0.661
	joint	0.860±0.008	0.745±0.002	0.876	0.763	0.817±0.008	**0.696±0.005**	0.830	**0.709**

## Footnotes

1 Details of its statistics and how it was extracted can be found in a separate technical report [13].

2 Some exams contain more than one image per view as technologists may need to repeat an image or provide a supplemental view to completely image the breast in a screening examination.

3 The sizes and locations of the cropping window for each image are adjusted to contain as much of the breast tissue as possible using a method explained in the section 2.D of the technical report on the dataset [13].

4 In Section I.B of the Supplementary Material, we show additional results for a view-wise model in which the weights for all views and sides are tied.

5 To finetune a network pretrained on RGB images with grayscale images, we duplicate the grayscale images across the RGB channels.

6 More precisely, this difference in AUC can be explained by the fact that while adding or subtracting negative examples to the test population does not change the true positive rate, it alters the false positive rate. False positive rate is computed as a ratio of false positive and negative. Therefore, when adding easy negative examples to the test set, the number of false positives will be growing slower than the number of all negatives, which will lead to an increase in AUC. On the other hand, removing easy negative examples will have a reverse effect and the AUC will be lower.

7 The readers were shown the images and asked to give their assessment. We confirmed the correctness of the format in which they returned their answers but we did not provide them with feedback on the accuracy of their predictions.

8 Masses are defined as 3-dimensional space occupying lesion with completely or partially convex-outward borders. Calcifications are tiny specks of calcific deposits. An asymmetry is defined as a unilateral deposit of fibroglandular tissue that does not meet the definition of mass, i.e., it is an area of the fibroglandular tissue that is not seen on the other breast. Architectural distortion refers to a disruption of the normal random pattern of fibroglandular tissue with no definite mass visible.

9 As one breast had two types of findings, the numbers add up to 63, not 62.

10 We do not have a way to tune *λ* to individual readers, hence we chose *λ* = 0.5 as the most natural way of aggregating two sets of predictions when not having prior knowledge of their quality. As Fig. 9 shows, an optimal *λ* varies a lot depending on the reader. The stronger the reader’s performance the smaller the optimal weight on the model. Notably though all readers can be improved by averaging their predictions with the model for both metrics.
